# A case report of neonatal adrenocortical carcinoma

**DOI:** 10.1186/1687-9856-2015-S1-P42

**Published:** 2015-04-28

**Authors:** Bui Phuong Thao, Vu Chi Dung, Nguyen Ngoc Khanh, Can Thi Bich Ngoc, Dang Anh Duong, Tran Ngoc Son, Hoang Ngoc Thach

**Affiliations:** 1 Vietnam National Hospital of Pediatrics, Hanoi, Vietnam

## 

Adrenocortical carcinoma is rarely seen in a neonatal period. Adrenocortical carcinoma usually causes virilisation, precocious puberty, Cushingoid syndrome.

## Aims

Describe a case of neonatal adrenocortical carcinoma diagnosed and treated in Vietnam National Hospital of Pediatrics.

## Method

A case report of neonatal adrenocortical carcinoma with Cushingoid syndrome and persistent hypertension.

## Results

A boy was admitted to hospital at the age of 23 days because of vomiting, poor feeding and abdominal distension, and edema on both legs. On examination we found the child had Cushingoid syndrome, but not precocious puberty, edema on both legs, and hypertenstion. Blood pressure was 150/90 mmHg, required IV Loxen to maintain BP. Investigation showed cortisol 8AM was high of 4473 nmol/l; cortisol 24AM 3971 nmol/l. Electrolyte, renal function, glucose, urine VMA/HMA were normal. Abdominal ultrasound found a hypo-enhancing mass in the left adrenal area with calcification and dimension of 37x32 mm. Dilated left renal pelvis was also observed. Cardiac ultrasound found ventricular hypertrophy, suspected cardiomyopathy. Neck MRI detected abnormal connection between lymphatic vessels and venous vessels. Abdominal CT showed a heterogeneous mass of 40x41x50 mm with calcification in the left adrenal area. Operation was done to remove the mass. No metastase was noticed during operation. Histology confirmed a diagnosis of adrenocortical carcinoma. Cortical was normal after operation. However, hypertension was still present a week after operation and IV Loxen was indicated to maintain normal BP.

## Conclusion

Post-operation hypertension was persistent in a neonatal adrenocortical carcinoma patient.

Written informed consent was obtained from the patient for publication of this Case report (and any accompanying images). A copy of the written consent is available for review by the Editor-in-Chief of this journal.

